# Disrupted in schizophrenia 1 and synaptic function in the mammalian central nervous system

**DOI:** 10.1111/ejn.12500

**Published:** 2014-04-08

**Authors:** Andrew D Randall, Mai Kurihara, Nicholas J Brandon, Jon T Brown

**Affiliations:** 1 School of Physiology and Pharmacology, School of Medical Sciences, University of BristolBristol, UK; 2 Institute of Biomedical and Clinical Sciences, University of ExeterThe Hatherley Building, Prince of Wales Road, Exeter, EX4 4PS, UK; 3 AstraZeneca Neuroscience iMEDCambridge, MA, USA

**Keywords:** elecrtophysiology, neurophysiology, psychiatric genetics, synaptic physiology, synaptic plasticity

## Abstract

The disrupted in schizophrenia 1 (DISC1) gene is found at the breakpoint of an inherited chromosomal translocation, and segregates with major mental illnesses. Its potential role in central nervous system (CNS) malfunction has triggered intensive investigation of the biological roles played by DISC1, with the hope that this may shed new light on the pathobiology of psychiatric disease. Such work has ranged from investigations of animal behavior to detailed molecular-level analysis of the assemblies that DISC1 forms with other proteins. Here, we discuss the evidence for a role of DISC1 in synaptic function in the mammalian CNS.

## Introduction

The underpinnings of many mental illnesses are multifactorial, arising from a combination of genetic susceptibilities and environmental factors (Tsuang, 2000; Moffitt *et al*., 2005; van Os *et al*., 2010). In psychiatric disease, it is therefore essential to develop an integrated understanding of the etiopathology, genetics and resultant modifications to key brain circuitry to aid our future battles to alleviate, or eliminate, patient suffering with novel therapeutic approaches. The research community is fortunate to have a growing methodological armory with which to fight these battles, including technological advances such as next-generation sequencing (Williams *et al*., 2009), alongside an ever-expanding experimental toolkit for functionally dissecting brain circuitry (Arenkiel & Ehlers, 2009).

The disrupted in schizophrenia 1 (DISC1) gene was originally identified in a unique Scottish pedigree, in which the gene is disrupted by an inherited balanced chromosomal translocation between chromosomes 1 and 11 [t(1;11)(q42.1;q14.3)]. Within this family, segregation is observed between the translocation event and a spectrum of psychiatric disorders, including major depression, schizophrenia, and bipolar disease (Jacobs *et al*., 1970; St Clair *et al*., 1990; Millar *et al*., 2000; Blackwood *et al*., 2001; Muir *et al*., 2008; Thomson *et al*., 2013). The molecular consequences of the translocation are likely to be very complex, as transcripts and abnormal proteins resulting from fusions between *DISC1* and a gene on chromosome 11, known as *Boymaw* or *FP1*, have recently been identified (Brandon & Sawa, 2011; Eykelenboom *et al*., 2012). To date, however, the focus has been largely restricted to the consequences of DISC1 truncation alone rather than functional outcomes related to translocation-generated fusion proteins. Expression of *DISC1* is also complex, and a striking number of splice forms (> 50) have been described (Nakata *et al*., 2009; Thomson *et al*., 2013). Which transcripts are actually translated into proteins is, however, not as yet fully understood.

Full-length human *DISC1* encodes an intracellular protein consisting of 854 amino acids with a molecular mass of ˜100 kDa. There is good evidence that the protein forms oligomeric species (Brandon *et al*., 2004; Narayanan *et al*., 2011), and a self-association domain has been identified biochemically (Kamiya *et al*., 2005). Furthermore, it has been proposed that assembly of DISC1 into large aggregates may play a role in its disease biology (Korth, 2012). This idea has been supported by the description of DISC1 aggresomes, which are intracellular entities that negatively impact on cellular transport processes (Atkin *et al*., 2011).

DISC1 expression is highest during central nervous system (CNS) development in both humans and rodents, and gradually decreases during life (Austin *et al*., 2004; Nakata *et al*., 2009). In adult mice, expression is seen in a broad range of brain areas, including the olfactory bulb, cortex, hippocampus, hypothalamus, cerebellum, and brainstem (Schurov *et al*., 2004). In some CNS areas, expression of *DISC* mRNA is seen only during development, with little if any mRNA being detectable in adulthood. Examples of such brain areas include the bed nucleus of the stria terminalis and the reticular thalamic nucleus (Austin *et al*., 2004). Although some earlier reports suggested that DISC1 was predominantly expressed in neurons and was largely absent from glia, more recent work has indicated that DISC1 expression may also occur in multiple classes of glial cell in both rodent and human tissue (Seshadri *et al*., 2010; Kuroda *et al*., 2011; Ma *et al*., 2013). Indeed, DISC1 has been implicated in cellular functions of both oligodendrocytes and astrocytes (Katsel *et al*., 2011; Ma *et al*., 2013).

As recently discussed elsewhere (Brandon & Sawa, 2011), despite its name, ‘disrupted in schizophrenia 1’, DISC1 may not be a key risk factor for schizophrenia. Instead, DISC1 disruption may confer a genetic risk at the level of endophenotypes or brain circuitry that underlies a number of major mental disorders. Indeed, at the genetic level, risk variants shared between multiple psychiatric disorders, such as schizophrenia, bipolar disorder, and autism, have been identified (Owen *et al*., 2007, 2011; Cuthbert & Insel, 2010). Important processes from where endophenotypes spanning a range of psychiatric disease might arise include the initial development of the nervous system and its subsequent refinement in the early years of life, and the functionality of synaptic connections within the circuits of the CNS. Both of these factors have been strongly implicated in the biology of DISC1 (Brandon & Sawa, 2011). This short review will specifically consider the latter, namely DISC1’s roles in synaptic function, although it must always be borne in mind that certain DISC1-related functional alterations identified at synapses might have their genesis in the prior developmental program of the CNS, rather than reflecting a specific ongoing action of the protein in adult synaptic functionality.

Although much about the biology and pathophysiology of DISC1 still remains elusive, it is well established that its major role is as a scaffold protein, serving to co-locate other important signaling molecules and place them in the necessary cellular locations for them to perform their various roles (Brandon, 2007; Thomson *et al*., 2013). Within the neuronal population, DISC1 expression occurs in multiple cell types, including both glutamatergic and GABAergic cells. Furthermore, DISC1 can be found in multiple cellular compartments, and appears to perform functions at multiple cellular loci, e.g. the nucleus, the centrosome, the primary cilium, and the mitochondrion (Brandon & Sawa, 2011; Thomson *et al*., 2013).

In support of roles related to synaptic functionality, DISC1 is highly enriched in postsynaptic density (PSD) fractions, and has been also shown to be present in dendritic spines by the use of ultrastructural methods (Kirkpatrick *et al*., 2006; Clapcote *et al*., 2007; Hayashi-Takagi *et al*., 2010; Carlisle *et al*., 2011; Wang *et al*., 2011; Paspalas *et al*., 2013). Furthermore, characterisation of the DISC ‘interactome’ has identified a number of proteins with known roles in the functional biology of synapses (Camargo *et al*., 2007). These interacting proteins include molecules that are themselves highly concentrated in PSD fractions, e.g. Traf2 and NcK-interacting kinase (Camargo *et al*., 2007; Wang *et al*., 2011).

So how does one set about investigating the potential roles of DISC1 in synaptic function? Unlike workers studying other proteins with pivotal roles in synaptic physiology, including receptors, ion channels, transporters, and enzymes such as kinases; the DISC1 investigator is not much helped by pharmacology. As DISC1 is a scaffold protein, there are no drugs that specifically inhibit or activate DISC1 function. Thus, recording synaptic function and testing the outcome of acutely changing DISC1 function pharmacologically is not currently an approach available to the DISC1 investigator. Instead, most investigations have relied on molecular manipulations that, in some way, modify the amount or type of DISC1 expressed in rodent tissues.

Manipulations used to modify DISC expression include the use of transgenic mice that express various forms of DISC1. These include mice in which various genetic engineering strategies have been employed to express truncation mutants designed to approximate the translocation found in the Scottish pedigree (Hikida *et al*., 2007; Pletnikov *et al*., 2008; Shen *et al*., 2008). In one case, expression of truncated DISC1 is specifically directed to astrocytes (Ma *et al*., 2013). A mouse has also been produced with a deletion of exons 2 and 3; these exons encode motifs that are important for many key protein–protein interactions, and, consequently, this mouse may have lost many functions of DISC1 (Kuroda *et al*., 2011). It is also noteworthy that a number of strains of laboratory mice are incapable of making full-length DISC1, owing to a 25-bp deletion in exon 6, first identified in 129S6/SvEv; this results in a string of 13 novel amino acids followed by a premature stop codon within exon 7 (Clapcote & Roder, 2006; Koike *et al*., 2006; Kvajo *et al*., 2008; Ritchie & Clapcote, 2013). Crossing these mice into a line that produces full-length DISC1, C57BL/6J, allows comparisons between normal mice and those with truncated DISC1. A mouse that expresses a C-terminal fragment of DISC1 under the control of tamoxifen induction has also been used to probe aspects of DISC1 physiology (Li *et al*., 2007). In addition, two mouse lines with point mutations in DISC1 (L100P and Q31L) derived from ethyl-nitrosourea treatment (Clapcote *et al*., 2007) have been investigated in some detail.

In addition to the use of various genetically modified mice, DISC1 expression has been induced or suppressed with chemical transfection or viral transduction of DISC1 expression constructs, or various DISC1-directed RNA interference (RNAi) reagents. A recent methodological approach that has been exploited successfully in studies of DISC1 biology is *in utero* gene transfer. This has been used to both knock down DISC1 expression and to overexpress normal or truncated DISC1 during early CNS development (Kamiya *et al*., 2005; Meyer & Morris, 2009; Kubo *et al*., 2010; Niwa *et al*., 2010; Tomita *et al*., 2011; Maher & LoTurco, 2012). Given DISC1’s role as a scaffold protein, another molecular approach that has been employed has been to disrupt the interaction between DISC1 and some of its binding partners by overexpressing peptides designed to disrupt specific DISC1 interaction sites (GIRDIN, Traf2 and NcK-interacting kinase, Lis1, NDE1, and FEZ1) (Taya *et al*., 2007; Enomoto *et al*., 2009; Kang *et al*., 2011; Wang *et al*., 2011).

As described above, a number of studies have indicated that manipulating the levels or nature of expressed DISC1 produces a range of effects on the development of the CNS. Many of these actions are likely to involve DISC1 binding partners, such as NDEL1, FEZ1, and GIRDIN (also known as KIAA1212). The most prevalent observations involve modifications to neurogenesis, neuronal migration, and integration of neurons into the neuronal parenchyma of their final destination. The last of these results in subpopulations of slightly misplaced neurons and alterations in mature neuronal morphology, such as changes in process branching (Kamiya *et al*., 2005; Duan *et al*., 2007; Faulkner *et al*., 2008; Shen *et al*., 2008; Enomoto *et al*., 2009; Kim *et al*., 2009; Kang *et al*., 2011; Kvajo *et al*., 2011). Although these effects are clear-cut, on the whole their global consequences are relatively subtle, e.g. slightly enlarged ventricles, a degree of cortical thinning, and small reductions in cell counts of certain neuronal populations – many of these have parallels in the human schizophrenia literature. Certainly, gross brain anatomy is not overtly disturbed in mice that lack full-length DISC1, whether as a result of genetic modification (Hikida *et al*., 2007; Pletnikov *et al*., 2008; Shen *et al*., 2008; Kuroda *et al*., 2011), cross-breeding of common laboratory strains (Koike *et al*., 2006), or *DISC1* knockdown by *in utero* delivery of short hairpin RNA (Koike *et al*., 2006). Consequently, although interfering with DISC1 function produces developmental changes, well-established synaptic pathways still develop and appear to show fundamental aspects of synaptic function.

With regard to evidence for changes in synaptic connectivity, there are certainly reports of a reduction in dendritic spine density when DISC1 is manipulated. For example, in the ethyl-nitrosourea-derived Q31L and L100P point mutation mice, spine density was approximately 15–20% lower in both the hippocampus and the frontal cortex, the latter being paralleled by a decreased number of neurons and a propensity for the neuronal population to be located slightly deeper in the cortex (Clapcote *et al*., 2007). As well as changes to spine numbers, manipulating DISC1 has significant effects on spine size in cultured neurons. Short-term knockdown of *DISC1* (2 days) increases both the number of spines and their size, whereas continuing inhibition of DISC1 expression up to 6 days results in fewer and smaller spines (Hayashi-Takagi *et al*., 2010). This latter observation also appears to translate to the *in vivo* situation when small interfering RNA (siRNA) is injected into the medial prefrontal cortex of young rats (Hayashi-Takagi *et al*., 2010). Additional evidence suggests that this effect on spine size is mediated by limiting the actions of Kalirin7 on Rac1 (Hayashi-Takagi *et al*., 2010).

Electrophysiological recording still remains the gold standard methodology for investigating alterations in synaptic function and related network activities. So far, there have been a relatively small number of neurophysiological investigations of the functional impacts of manipulating DISC1. These data frequently appear as fractional parts of studies in which other experimental approaches are also employed. Certainly, so far, the neurophysiological literature related to DISC1 is in its infancy as compared with the in-depth work performed on many other models of CNS disease. For example, we are not aware of any published studies of DISC1-related electrophysiological work performed *in vivo*.

The reported concentration of DISC1 expression in the adult PSD fraction (Kirkpatrick *et al*., 2006; Clapcote *et al*., 2007; Hayashi-Takagi *et al*., 2010; Carlisle *et al*., 2011; Wang *et al*., 2011), along with the known functions of some of the proteins for which it acts as a scaffold (Camargo *et al*., 2007; Brandon & Sawa, 2011), have led most workers so far to focus their electrophysiological investigations on fast excitatory signaling mediated by postsynaptic ionotropic glutamate receptors. All such work to date has focused on recordings made in hippocampal or cortical neurons, either in brain slices or in primary cultures. Notably, the limited number of electrophysiological studies performed to date have employed tissue derived from a number of different models, each of which has different underlying genetics. For this reason, it is frequently hard to compare findings between groups, which can, in some cases, at first sight appear contradictory. We believe that, as more studies are performed in the future, the key DISC1-related neurophysiological phenotypes will become clearer, and the work performed to date will provide useful indicators as to what should be examined.

## Hippocampal electrophysiology

Comparatively high levels of DISC1 are expressed both in the dentate gyrus and in its major output region, the hippocampal CA3 subfield. Manipulation of DISC1 is known to produce effects on adult neurogenesis and wiring in this brain area (Duan *et al*., 2007; Faulkner *et al*., 2008; Kim *et al*., 2009). Dentate granule neurons, the principal cells of the dentate gyrus, receive their principle glutamatergic input from the axons of the perforant path arriving from the cerebral cortex. The axons of granule neurons are the mossy fibers, and these form synapses with various hippocampal neurons, including CA3 pyramidal cells, mossy cells of the hilus, and various GABAergic interneurons. Both the perforant path and mossy fiber projections have been investigated in mice expressing various forms of DISC1. Electrophysiological investigation of the medial perforant path was performed in brain slices from mice with targeted disruption of exons 2 and 3 (Kuroda *et al*., 2011). In these animals, there was no difference in the efficacy of basal synaptic transmission as determined by input–output curves obtained after single-shock stimulation of the perforant path. The same group also looked at the induction of long-term potentiation (LTP) in this pathway (Kuroda *et al*., 2011). This was inducible in the mice with the targeted deletion but may have been subtlety altered, although, notably, the group sizes employed were small (only *n* = 4 or *n* = 5), and LTP was also small in both genotypes, probably because the experiments were not performed with GABA receptor antagonists, which is the normal methodology employed for studying perforant pathway LTP in brain slices.

Mossy fiber transmission has been examined in hippocampal slices from DISC1^Tm1Kara^ mice, animals derived by breeding the natural truncation mutation first described in the 129S6/SvEv strain into a C57BL6 background (Koike *et al*., 2006). These mice show anatomical abnormalities in mossy fibers (Kvajo *et al*., 2011), which one might expect to be apparent in neurophysiological measurements. Input–output curves obtained with extracellular methods were unaltered in 6–8-week-old DISC1^Tm1Kara^ mice, as was mossy fiber LTP (Kvajo *et al*., 2011). However, the profound short-term synaptic plasticity shown by mossy fibers was reduced in the mutant line, as demonstrated by a range of measurements, including both paired-pulse and frequency facilitation (Kvajo *et al*., 2011). Our own studies of mossy fibers in DISC1_tr_ mice revealed an entirely different outcome. These animals transgenically express a bacterial artificial chromosome bearing a C-terminal truncated form of murine DISC1, the truncation being related to the putative translocation-mediated truncation in the Scottish family (Shen *et al*., 2008). DISC1_tr_ mice have a small (˜20%) enhancement of mossy fiber paired-pulse facilitation at all interstimulus intervals examined, but no change in 1-Hz frequency facilitation studied with 20 consecutive stimuli (M. Camo, J. Brown and A. Randall, unpublished observations). Kvajo *et al*. (2011) also examined the intrinsic excitability properties of dentate granule cells in slices from 4–6-week-old mice. They saw no change in resting potential, but identified a significant decrease in input resistance of ˜20%. When the ability of depolarising current stimuli to elicit action potential firing was investigated, the decrease in excitability and increase in rheobase expected to accompany a decrease in input resistance were both observed (Kvajo *et al*., 2011). Thus, truncation of DISC seems to be able to affect both the intrinsic excitability of, and synaptic output from, dentate granule cells.

Hippocampal CA1 pyramidal cells very probably constitute the most widely studied class of neuron in the mammalian CNS. Furthermore, the input to these cells from CA3 pyramidal cells, the Schaffer collateral commissural pathway (SCCP), is probably the most widely studied single pathway in the mammalian brain. Synaptic transmission in this pathway was investigated in mice engineered to express a C-terminal fragment of DISC1 in the forebrain in an inducible and reversible fashion. These animals, which showed a number of behavioral changes when expression of the DISC1 fragment was induced, also had a depressed input–output relationship in the SCCP at the age of 3–4 months. However, no changes in either short-term plasticity or LTP were found (Li *et al*., 2007).

The SCCP has also been studied in DISC1^Tm1Kara^ mice. Extracellular recordings performed in transverse slices from 7–9-week-old animals revealed no genotype dependence of input–output relationships or paired-pulse facilitation. LTP induced with two repeats of a 100-Hz, 1-s conditioning stimulus was also not altered; however, a small decrease in the excitatory postsynaptic potential potentiation seen in the minutes following application of the conditioning stimulus was described (Kvajo *et al*., 2008). Our own DISC1-related study of the neurophysiology of hippocampal area CA1 is included elsewhere in this issue (Booth *et al*., 2014). Like our unpublished work on mossy fibers mentioned above, these studies used the DISC1_tr_ mouse, which expresses a bacterial artificial chromosome bearing a C-terminal truncated form of DISC1. We used a combination of whole cell patch clamp recording and extracellular methods to study synaptic function in both the SCCP and the temporoamonic input to CA1 pyramidal cells (the latter pathway is the direct, monosynaptic input from the entorhinal cortex to CA1 pyramidal neurons). In the DISC1_tr_ cohort, neither pathway showed changes in input–output relationships or short-term synaptic dynamics. Also, the ratio of *N*-methyl-d-aspartate (NMDA) receptor-mediated and AMPA receptor-mediated components of the synaptic response was not genotype-dependent. Interestingly, however, we found a clear enhancement of theta burst-induced LTP in the SCCP of DISC1_tr_ mice, but a complete loss of LTP in the temporoamonic pathway. The mechanisms underpinning these pathway-specific effects of the truncated form of DISC1 on long-term synaptic plasticity require further investigation. Our article also describes our single-cell recordings used to compare a broad range of intrinsic excitability properties in CA1 pyramidal cells from 4-month-old DISC1_tr_ mice and wild-type littermates. These identified no substantial dependence on genotype.

One report concerning DISC1 function in hippocampal neurons describes the use of mixed hippocampal cultures prepared from embryonic day 17.5 mouse embryos and grown on multielectrode arrays (MacLaren *et al*., 2011). This group examined the effects on network activity of siRNA-mediated knockdown of four genes implicated in schizophrenia, including *DISC1*. These experiments suggested that reducing the level of DISC1 by ˜60% may serve to increase the duration of action potential bursts, although whether this reflects an intrinsic or synaptic change is not apparent from this type of data.

## Electrophysiology in the cerebral cortex

Beyond the hippocampus, DISC1 neurophysiology has been studied in various preparations of the rodent cerebral cortex. In a similar knockdown approach to that employed by MacLaren *et al*. (2011) with hippocampal cultures, the effects of *DISC1* knockdown were examined in cortical cultures as part of a study predominantly focused on the role of DISC1 in the regulation of spine properties by Kalirin7 and Rac1 (Hayashi-Takagi *et al*., 2010). *DISC1* knockdown increased the frequency of miniature excitatory postsynaptic currents (EPSCs) by ˜30%, probably reflecting the increased number of spines present under these conditions. Cortical cultures have also been employed to demonstrate that knockdown of *DISC1* with siRNA increases NMDA receptor-mediated currents in response to exogenous agonist application (Wei *et al*., 2014). This effect was reported to arise from increased surface expression of the GluN2A subunit. The same authors also used whole cell recordings from slices of rat prefrontal cortex following prior *in vivo* injection of lentivirus producing *DISC1* short hairpin RNA (Wei *et al*., 2014). Examining electrically evoked, isolated NMDA receptor-mediated EPSCs at positive membrane potentials, they found that amplitudes were enhanced by approximately 30–55% in response to *DISC1* knockdown, in line with their findings in cultures. This enhancement *in vivo*, as in cultures, seemingly arose through a greater contribution from NR2A receptors (Wei *et al*., 2014).

Other neurophysiological studies in the cortex have also employed brain slices. One study examined slices from two different mouse models – the first was the DISC1^Tm1Kara^ mouse line described above (Koike *et al*., 2006), and the second was the line expressing a truncated human DISC1 construct in a forebrain-specific manner, developed by Pletnikov *et al*. (2008). In this latter study (Holley *et al*., 2013), spontaneously arising excitatory and inhibitory events in layer 2/3 of the prefrontal cortex in mice aged ˜5 months were analysed in both sexes. The data obtained pointed towards an increased frequency of EPSCs in both mouse lines, although the changes were relatively modest. In our own studies, we have seen decreased spontaneous synaptic activity in layer V neurons of the medial prefrontal cortex of DISC1_tr_ mice, although intrinsic excitability properties of layer five neurons were very slightly decreased (M. Kurihara, J. Brown and A. Randall, unpublished observations).

Acutely prepared slices of mouse prefrontal cortex have also been used to study the neurophysiological consequences of *in utero* knockdown of DISC1 (Niwa *et al*., 2010). Layer 2/3 cells that had received the RNAi construct (as revealed by green fluorescent protein labeling) showed no change in resting potential, but had a higher membrane resistance and a lower capacitance, both of which are likely to be consequences of the impaired dendritic development shown by these cells. In contrast, deep layer cortical neurons, which are not targeted by *in utero* electroporation, have unaltered membrane resistance or membrane potential. However, dopamine D2ergic modulation of electrically evoked excitatory postsynaptic potentials in these deep layer neurons is strongly attenuated when *DISC1* is knocked down. This observation is thought to be related to the disturbed dopaminergic innervation of this cortical area produced by the knockdown of *DISC1* (Niwa *et al*., 2010).

DISC1 levels are unquestionably high in postsynaptic elements. Additionally, a number of DISC1 binding partners also show prominent postsynaptic localisation and possess known postsynaptic roles. Consequently, investigations of DISC1 neurophysiology at synapses have tended to concentrate on postsynaptic functionality. However, DISC1 expression appears also to be a feature of some axons and presynaptic terminals (Kirkpatrick *et al*., 2006; Wang *et al*., 2011). Furthermore, DISC1 also interacts with proteins with known roles in functional presynaptic physiology (Camargo *et al*., 2007), and perturbations to DISC1 modify axonal development (Duan *et al*., 2007; Faulkner *et al*., 2008; Kvajo *et al*., 2008; Enomoto *et al*., 2009). Together, these observations indicate that DISC1 may also have roles in presynaptic neurophysiologal activities, including neurotransmitter release. This idea is supported by the changes in short-term plasticity in mossy fibers that we highlighted above (Kvajo *et al*., 2011; M. Camo, J. Brown and A. Randall, unpublished observations), as this form of synaptic plasticity is well known to be an entirely presynaptic phenomenon.

A recent elegant study using optogenetic methodologies has provided more direct evidence for presynaptic functions of DISC1 in the neocortex of Wistar rats (Maher & LoTurco, 2012). The authors used *in utero* electroporation to introduce Cre-dependent inducible expression vectors for full-length DISC1 or a C-terminal truncation of DISC1 or DISC1 RNAi reagents, in addition to channelrhodopsin2. This permitted both expression of both DISC1-related contructs and light-mediated activation to be confined entirely to presynaptic elements. Recording from layer 2/3 cells, the authors found that spontaneous EPSC frequencies were enhanced by a truncated form of DISC1 (Maher & LoTurco, 2012), a finding in agreement with the observations of Holley *et al*. (2013) in transgenic mouse lines. Using brief light pulses to activate channelrhodopsin2, and thereby trigger evoked neurotransmitter release, the authors also found that expression of the truncated DISC1 construct interfered with the kinetics of glutamate release. Using paired light pulses, they also demonstrated that wild-type DISC1 seems to modify release probability, such that more DISC1 increases the probability of release, whereas *DISC1* knockdown lowers the probability of release. In future, it will be interesting to determine which proteins DISC1 is interacting with to produce these actions in presynaptic elements. Possibly related to these functional observations of altered presynaptic function is the observation that full-length DISC1 promotes, and C-terminally truncated DISC1 retards, transport along neuronal processes of vesicles containing a synaptic vesicle protein target (Flores *et al*., 2011).

## Conclusion

In conclusion, neurophysiological investigation of the neurobiological functions of DISC1 is still in its infancy. We believe that further analyses using neurophysiological measures will provide important insights into the biology of this protein and, consequently, pathways with important roles in psychiatric disease. The data produced to date indicate that alterations to DISC1, such as those that elicit psychiatric disease, can affect multiple processes. As described above, there is evidence for activities on both the presynaptic and postsynaptic sides of the fast chemical synapse, and for interactions with processes controlling long-term synaptic plasticity. The first hints of how these might impact on the behavior of intact complex networks are appearing, although there is a clear need for suitable *in vivo* studies in appropriate models. Such work is currently ongoing in our group and presumably elsewhere as well, so we should expect to see data on *in vivo* DISC1-related neurophysiology in the near future.

## Abbreviations

CNS: Central nervous system
DISC1: disrupted in schizophrenia 1
EPSC: excitatory postsynaptic current
LTP: long-term potentiation
NMDA: *N*-methyl-d-aspartate
PSD: postsynaptic density
RNAi: RNA interference
SCCP: Schaffer collateral commissural pathway
siRNA: small interfering RNA
